# Editorial: Tendon Structure-Function Relationship in Health, Ageing, and Injury

**DOI:** 10.3389/fspor.2021.701815

**Published:** 2021-06-18

**Authors:** Huub Maas, Toni Arndt, Jason R. Franz

**Affiliations:** ^1^Department of Human Movement Sciences, Faculty of Behavioural and Movement Sciences, Vrije University Amsterdam, Amsterdam Movement Sciences, Amsterdam, Netherlands; ^2^Swedish School of Sport and Health Sciences, Stockholm, Sweden; ^3^University of North Carolina at Chapel Hill and North Carolina State University, Chapel Hill, NC, United States

**Keywords:** subtendon, non-uniform deformation, Achilles tendon, ultrasound, aging, skeletal muscle

Tendons connect muscle fibers to the skeleton and, thereby, serve as transmitters of muscle force. Through their viscoelastic properties, tendons can store and release energy during movement contributing to the performance of motor tasks including locomotion. In contrast to the representation of tendons in textbooks and models of the musculoskeletal system, tendons are not simple viscoelastic bands, but are intricate multi-stranded structures. The objective of this Research Topic was to bring together studies aimed at improving our understanding of the tendon structure-(dys)function relationship in health and disease, with a particular interest in tendons linked to multiple muscles. All but one (Audenaert et al.) of the contributions investigated the Achilles tendon (AT), which is contiguous with the soleus and lateral and medial gastrocnemius muscles. Several other tendons arise from multiple muscles, such as the patellar tendon, and would be equally interesting to investigate.

Using a simulation approach, enhanced lengthening of the M. Psoas major tendon was found to be related to a rotational variant in human femoral morphology (Audenaert et al.). The influence of the skeletal system on AT mechanical properties was also indicated as a factor explaining the lack of tendon adaptation to mechanical loading during growth in a guinea fowl model (Katugam et al.). These studies exemplify the importance of assessing tendon behavior and adaptation within an integrated framework, such as described by Pizzolato et al.. Ultrasound imaging has been frequently used to study the differences in displacement of the deep and superficial portions of the AT (e.g., Arndt et al., 2012; Slane and Thelen, 2014). Internal tendon tissue displacements were more uniform when an ankle foot orthosis was applied and when the range of motion was reduced (Froberg et al.). A simulation study also reported that the amount of sliding between subtendons was task specific (Handsfield et al.). They further found effects of subtendon twisting and connectivity between subtendons. Tendon tissue displacements were found to be less uniform when subtendons were substantially twisted (Knaus and Blemker). Important for the ultrasound-based assessment of non-uniform displacements, a twisted morphology of the AT was also found to result in errors when estimating local strains. Regarding the connectivity between subtendons, clear evidence of force transmission via the inter-subtendon matrix was provided for rat AT (Gains et al.). Previous studies have demonstrated effects of aging on the mechanical properties of tendon (Sprague et al., 2020), its matrix (Thorpe et al., 2015) and non-uniformity of tendon tissue displacements (Franz and Thelen, 2015). In this Research Topic, it was shown that the structure-function relationship between the AT and the triceps surae muscles is disrupted in older adults (Knaus et al.). Using shear wave tensiometry to assess AT forces, differential effects of age on work performed by the soleus and gastrocnemius were observed (Ebrahimi et al.). While this method does not allow assessment of forces of each of the subtendons, such effects of age may also be related to the changes in mechanical independence between subtendons.

It is evident from the articles in this topic that the notion of subtendons in the human Achilles tendon, as introduced recently (Handsfield et al., 2016), has become relatively accepted terminology in the field of biomechanics, including some of the editors' own work. Nevertheless, the editors of this topic feel called to provide a note of caution concerning any definitive determination about the division of the human AT into subtendons and their relative independence. Studies on subtendons in the rat AT convincingly demonstrate that these are at least not purely independent structures (e.g., Finni et al., 2018). Previous studies and those presented in this Research Topic are providing solid evidence that behavior, in particular internal tendon tissue displacements, differs across separate regions of the tendon and that this correlates in anatomically consistent ways with individual muscle length changes. An important question raised is whether the relatively clear identification of inter-subtendon regions in rat and rabbit AT (Gains et al.; Handsfield et al.) can also be applied to human AT. Although our interpretations often consider the possibility, it is not yet clear that these regions can be specifically attributed to individual muscles—a prerequisite to the premise upon which subtendons are based. Anatomic human cadaver analysis shows that the collagenous material of the distal AT cannot be easily dissected into subtendons (Arndt et al., 1997) without considerable force. Recent evidence from ultrasound speckle tracking studies shows that adjacent fascicles within the tendon can slide relative to each other (e.g., Arndt et al., 2012; Slane and Thelen, 2014). However, such sliding appears to be a phenomenon occurring throughout the tendon's cross-sectional area. More sophisticated imaging or analysis routines (see for example Handsfield et al.) are warranted to investigate the extent to which boundary regions exist within longitudinal tendon tissue displacement fields that could signal the presence or refute the premise of individual subtendons, *per se*. We therefore suggest that caution be exercised and the state of the science be acknowledged when using the term subtendon to investigate and/or describe internal human AT tissue function.

Sliding within the AT may be necessary to allow muscles crossing one (soleus) or two joints (gastrocnemius) with distinct activation patterns (Moritani et al., 1991) to function normally, and lack of sliding may be a sign of malfunction (Franz and Thelen, 2015; Froberg et al., 2017). For rat AT, it was described that while the elastic interfascicular matrix allows relative sliding of the tendon fascicles (Finni et al., 2018), it can also bear considerable loads (see also Gains et al.). Non-uniform fascicle sliding may facilitate protective mechanisms for tendon sections subjected to greater strain, thus preventing excessive forces in the relevant fascicles. Consequently, tendon organization may provide an inbuilt protective mechanism against tendon injuries (Maas and Finni, 2018) and lack of sliding may be a risk factor for injury or disease (Franz and Thelen, 2015; Froberg et al., 2017). Ultimately, research on tendons arising from multiple muscles should continue to elucidate the role of maintaining non-uniform tendon tissue displacements and its implications for injury, pathology, aging, and rehabilitation.

## Author Contributions

All authors listed have made a substantial, direct and intellectual contribution to the work, and approved it for publication.

## Conflict of Interest

The authors declare that the research was conducted in the absence of any commercial or financial relationships that could be construed as a potential conflict of interest.

## References

[B1] Arndt A. Bengtsson A. S. Peolsson M. Thorstensson A. Movin T. (2012). Non-uniform displacement within the Achilles tendon during passive ankle joint motion. Knee Surg. Sports Traumatol. Arthrosc. 20, 1868–1874. 10.1007/s00167-011-1801-9 22120840

[B2] Arndt A. Notermans H.-P. Koebke J. Bruggemann G. P. (1997). Mazeration der menschlichen Achillessehne zur Betrachtung der kollagenen Fasern. Der. Präparator. 43, 63–70.

[B3] Finni T. Bernabei M. Baan G. C. Noort W. Tijs C. Maas H. (2018). Non-uniform displacement and strain between the soleus and gastrocnemius subtendons of rat Achilles tendon. Scand. J. Med. Sci. Sports 28, 1009–1017. 10.1111/sms.13001 29094399

[B4] Franz J. R. Thelen D. G. (2015). Depth-dependent variations in Achilles tendon deformations with age are associated with reduced plantarflexor performance during walking. J. Appl. Physiol. 119, 242–249. 10.1152/japplphysiol.00114.2015 26023223 PMC4526706

[B5] Froberg A. Cisse A. S. Larsson M. Martensson M. Peolsson M. Movin T.. (2017). Altered patterns of displacement within the Achilles tendon following surgical repair. Knee Surg. Sports Traumatol. Arthrosc. 25, 1857–1865. 10.1007/s00167-016-4394-5 28004174 PMC5487597

[B6] Handsfield G. G. Slane L. C. Screen H. R. (2016). Nomenclature of the tendon hierarchy: an overview of inconsistent terminology and a proposed size-based naming scheme with terminology for multi-muscle tendons. J. Biomech. 49, 3122–3124. 10.1016/j.jbiomech.2016.06.028 27421207

[B7] Maas H. Finni T. (2018). Mechanical coupling between muscle-tendon units reduces peak stresses. Exerc. Sport Sci. Rev. 46, 26–33. 10.1249/JES.0000000000000132 28857890

[B8] Moritani T. Oddsson L. Thorstensson A. (1991). Phase-dependent preferential activation of the soleus and gastrocnemius muscles during hopping in humans. J. Electromyogr. Kinesiol. 1, 34–40. 10.1016/1050-6411(91)90024-Y 20719593

[B9] Slane L. C. Thelen D. G. (2014). Non-uniform displacements within the Achilles tendon observed during passive and eccentric loading. J. Biomech. 47, 2831–2835. 10.1016/j.jbiomech.2014.07.032 25150898 PMC4163107

[B10] Sprague A. L. Awokuse D. Pohlig R. T. Cortes D. H. Silbernagel K. G. (2020). Relationship between mechanical properties (shear modulus and viscosity), age, and sex in uninjured Achilles tendons. Transl. Sports Med. 3, 321–327. 10.1002/tsm2.148 33196016 PMC7665100

[B11] Thorpe C. T. Godinho M. S. Riley G. P. Birch H. L. Clegg P. D. Screen H. R. (2015). The interfascicular matrix enables fascicle sliding and recovery in tendon, and behaves more elastically in energy storing tendons. J. Mech. Behav. Biomed. Mater. 52, 85–94. 10.1016/j.jmbbm.2015.04.009 25958330 PMC4655227

